# Predicting Metabolic Pathways of Small Molecules and Enzymes Based on Interaction Information of Chemicals and Proteins

**DOI:** 10.1371/journal.pone.0045944

**Published:** 2012-09-21

**Authors:** Yu-Fei Gao, Lei Chen, Yu-Dong Cai, Kai-Yan Feng, Tao Huang, Yang Jiang

**Affiliations:** 1 Department of Surgery, China-Japan Union Hospital of Jilin University, Changchun, China; 2 College of Information Engineering, Shanghai Maritime University, Shanghai, China; 3 Institute of Systems Biology, Shanghai University, Shanghai, China; 4 Beijing Genomics Institute, Shenzhen Beishan Industrial zone, Shenzhen, China; 5 Key Laboratory of Systems Biology, Shanghai Institutes for Biological Sciences, Chinese Academy of Sciences, Shanghai, China; 6 Shanghai Center for Bioinformation Technology, Shanghai, China; Russian Academy of Sciences, Institute for Biological Instrumentation, Russian Federation

## Abstract

Metabolic pathway analysis, one of the most important fields in biochemistry, is pivotal to understanding the maintenance and modulation of the functions of an organism. Good comprehension of metabolic pathways is critical to understanding the mechanisms of some fundamental biological processes. Given a small molecule or an enzyme, how may one identify the metabolic pathways in which it may participate? Answering such a question is a first important step in understanding a metabolic pathway system. By utilizing the information provided by chemical-chemical interactions, chemical-protein interactions, and protein-protein interactions, a novel method was proposed by which to allocate small molecules and enzymes to 11 major classes of metabolic pathways. A benchmark dataset consisting of 3,348 small molecules and 654 enzymes of yeast was constructed to test the method. It was observed that the first order prediction accuracy evaluated by the jackknife test was 79.56% in identifying the small molecules and enzymes in a benchmark dataset. Our method may become a useful vehicle in predicting the metabolic pathways of small molecules and enzymes, providing a basis for some further analysis of the pathway systems.

## Introduction

Metabolism defines a series of chemical reactions that occur in a cell, maintaining the lives of living organisms by supplying the necessary molecules and energy [1]. Metabolism is composed of metabolic pathways in which chemical reactions are organized in such a way that one molecule is transformed into another through a cascade of reactions recruiting small molecules and enzymes. Thus, small molecules and enzymes are part of the basic components of metabolic pathways. Determining the functioning of the small molecules and enzymes of each metabolic pathway is key to understanding the metabolic pathway and its biological functions.

During the past decade, large amounts of information concerning different organisms have been gathered on both the genetic and metabolic levels. Some databases pertaining to chemicals and proteins, such as KEGG (Kyoto Encyclopedia of Genes and Genomes) [2], [3], ENZYME [4], STITCH (Search Tool for Interactions of Chemicals) [5] and STRING (Search Tool for the Retrieval of Interacting Genes/Proteins) [6], have been established, from which descriptions of the properties of small molecules and enzymes can be readily acquired. Such information also provides an opportunity to study the metabolic pathways in greater detail computationally. A computational approach is another avenue by which to gain insight into metabolic pathways, apart from biochemical experiments. In recent years, some efforts [1], [7], [8] have been made to tackle the problem by mapping small molecules to the corresponding metabolic pathways. However, besides small molecules, enzymes are also important basic components of metabolic pathways. As far as we know, this study is the first to map small molecules and enzymes to the metabolic pathways simultaneously, thus providing some additional information for use in studying metabolic pathways.

**Figure 1:**
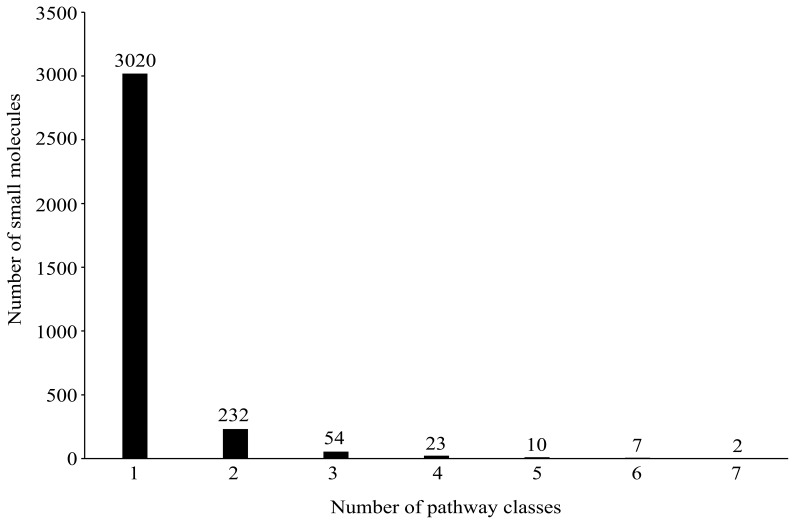
The number of small molecules against the number of pathway classes.

**Table 1:** Distribution of 3,348 small molecules and 654 enzymes of yeast in the 11 metabolic pathway classes.

Tag	Metabolic pathway class	Number of small molecules	Number of enzymes	Total
*M*_1_	Carbohydrate Metabolism	394	198	592
*M*_2_	Energy Metabolism	151	146	297
*M*_3_	Lipid Metabolism	399	84	483
*M*_4_	Nucleotide Metabolism	133	107	240
*M*_5_	Amino Acid Metabolism	489	158	647
*M*_6_	Metabolism of Other Amino Acids	156	44	200
*M*_7_	Glycan Biosynthesis and Metabolism	47	18	65
*M*_8_	Metabolism of Cofactors and Vitamins	350	87	437
*M*_9_	Metabolism of Terpenoids and Polyketides	507	18	525
*M*_10_	Biosynthesis of Other Secondary Metabolites	509	17	526
*M*_11_	Xenobiotics Biodegradation and Metabolism	709	21	730
Total	–	3,844	898	4,742

A large body of data concerning protein-protein interactions and chemical-chemical interactions has been applied extensively to predicting the attributes of proteins and compounds [8], [9], [10], [11], [12], [13], [14]. This work led to the conclusion that interactive proteins or interactive compounds were more likely to share common biological functions than non-interactive ones. Most of these approaches studied chemical-chemical interactions or protein-protein interactions separately to construct classification models. In this study, we proposed a novel method, integrating interactions among chemicals and proteins including chemical-chemical interactions, protein-protein interactions, and chemical-protein interactions, to predict metabolic pathways in which small molecules and enzymes of yeast participate. Since some small molecules and enzymes participate in more than one metabolic pathway, our method sorts the probabilities of metabolic pathways to which a small molecule or enzyme may belong rather than predicting only the most probable metabolic pathway.

## Materials and Methods

### Benchmark Dataset

The dataset of small molecules to be studied was downloaded from the FTP site of the public database KEGG [2], [3] at ftp://ftp.genome.jp/pub/kegg (June, 2011), from which we extracted 17,641 small molecules. After excluding small molecules that do not participate in any metabolic pathway, 4,487 small molecules were retained. The dataset of enzymes of yeast were also acquired from the FTP site of the public database KEGG [2], [3] at ftp://ftp.genome.jp/pub/kegg (November, 2010). Likewise, those enzymes that do not participate in any metabolic pathway were excluded. Thus, we retained 655 enzymes of yeast, whose data on participation in metabolic pathways is available.

**Figure 2:**
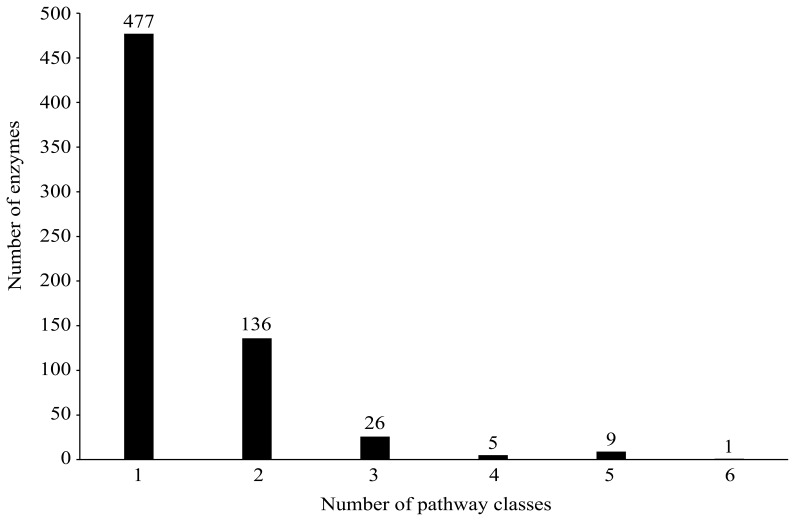
The number of enzymes against the number of pathway classes.

**Table 2:** The interactive compounds and proteins of C07277 and YLL058W.

Row index	Compound/Enzyme	Compound/Enzyme	Interaction confidence score	Tag of metabolic pathway class a
1	C07277	C00103	409	*M*_1_, *M*_9_, *M*_10_
2	C07277	C00363	441	*M*_4_
3	C07277	C00507	416	*M*_1_
4	C07277	C03319	446	*M*_9_, *M*_10_
5	C07277	C11912	63	*M*_9_
6	C07277	YDL055C	298	*M*_1_
7	YLL058W	C00087	317	*M*_2_
8	YLL058W	C00109	900	*M*_1_, *M*_5_
9	YLL058W	C00155	900	*M*_2_, *M*_5_
10	YLL058W	C00283	317	*M*_2_, *M*_5_
11	YLL058W	C00542	904	*M*_2_
12	YLL058W	C01077	900	*M*_2_, *M*_5_
13	YLL058W	C02291	900	*M*_5_
14	YLL058W	C05688	900	*M*_6_
15	YLL058W	C05699	900	*M*_6_
16	YLL058W	YAL012W	463	*M*_2_, *M*_5_
17	YLL058W	YGL184C	241	*M*_2_, *M*_5_

a The information in this column represents the metabolic pathway classes of the compound or enzyme in column 3.

As described above, 4,487 small molecules and 655 enzymes of yeast have recoverable information concerning their participation in metabolic pathways. These samples were used to comprise a dataset **S** ^ce^. However, not all samples can be used in our method due to the lack of interaction information. Those not having any interactions with other compounds or proteins in **S** ^ce^ were excluded. Finally, we obtained 4,002 samples including 3,348 small molecules and 654 enzymes, formulated by **S = S** *_c_*∪**S** *_e_*, where **S** denotes the benchmark dataset consisting of 4,002 samples, **S** *_c_* the dataset consisting of 3,348 small molecules, and **S** *_e_* the dataset set consisting of 654 enzymes.

According to KEGG (http://www.genome.jp/kegg/pathway.html), there exist more than 150 metabolic pathways, classified into 11 major metabolic pathway classes (see column 2 of **Table 1**). Subsequently, 3,348 small molecules and 654 enzymes were mapped into the 11 major metabolic pathway classes. The distribution of these small molecules and enzymes is shown in **Table 1**. The coding of small molecules and enzymes in each of the 11 major metabolic pathway classes can be found in Online Supporting Information S1. From column 3 of **Table 1**, the sum of small molecules in all pathways is greater than the total number of small molecules in the dataset, indicating that some small molecules belong to more than one pathway class. In detail, 3,020 small molecules belong to only one pathway class, while others belong to more than one pathway class - see **Figure 1** for the number of small molecules versus the number of pathway classes. Likewise, as given in column 4 of **Table 1**, some enzymes also appear in more than one pathway class. In detail, 477 enzymes appear in only one pathway class, while others appear in at least two pathway classes (see **Figure 2** for detail). In view of this, it appears to be a multi-label problem to predict the pathway classes of small molecules and enzymes. Similar to the cases in predicting some other attributes of proteins and compounds [11], [12], [15], [16], [17], the proposed method needs to provide a series of candidate pathway classes for a query small molecule or enzyme.

### Construction of Hybrid Network

It is known that interactive proteins and compounds are more likely to share common biological functions [8], [9], [12], [13], [14], [18] than would non-interactive ones; given a compound, its biological functions may share the same functions with its interactive proteins. Conversely, the biological functions of a protein may also be similar to the functions of its interactive compounds. In this case, if a compound and a protein are interactive with one another, it would be more likely that they appear in the same metabolic pathway. In view of this, a hybrid interaction network was constructed as follows.

The constructed network takes small molecules and enzymes as its nodes, and an edge is drawn between two nodes if and only if the corresponding small molecule and enzyme can interact with one another. Different combinations of the participants lead to three kinds of interactions: chemical-chemical interactions, chemical-protein interactions, and protein-protein interactions. The data concerning chemical-chemical interactions and chemical-protein interactions was acquired from STITCH (http://stitch.embl.de/) [5], a well-known database containing known and predicted interactions of chemicals and proteins derived from experiments, literature and other databases. To more accurately represent the interaction network, each edge in the network was labeled with a score given as the edge weight to quantify the interaction confidence, *i.e.*, the likelihood that an interaction may occur. For any two small molecules *c*_1_ and *c*_2_, their interaction confidence score, *i.e.*, the weight of the edge with *c*_1_ and *c*_2_ as endpoints, was denoted by Q*_cc_*(*c*_1_, *c*_2_). Specifically, if the interaction between *c*_1_ and *c*_2_ does not exist in STITCH, their interaction confidence score was set to 0, *i.e.*, Q*_cc_*(*c*_1_, *c*_2_) = 0. Likewise, the weight of the edge with one small molecule *c* and one enzyme *e* as endpoints was denoted by Q*_cp_*(*c*, *e*). In particular, the confidence score was set to be 0 if the interaction between *c* and *p* does not exist in STITCH. The data concerning protein-protein interactions was retrieved from STRING (http://string.embl.de/) [6], a large database containing known and predicted protein interactions including direct (physical) and indirect (functional) interactions that were derived from several sources such as experimental repositories and computational prediction methods. Like the previous case of chemical-chemical interactions and chemical-protein interactions, each edge with two proteins *p*_1_ and *p*_2_ as endpoints was labeled with a score, denoted by Q*_pp_*(*p*_1_, *p*_2_), to quantify the interaction confidence, *i.e.*, the likelihood that an interaction may occur. In particular, if *p*_1_ and *p*_2_ are non-interactive proteins according to the data in STRING, their interaction confidence score was set to 0, *i.e.*, Q*_pp_*(*p*_1_, *p*_2_) = 0.

**Table 3:** The likelihood of C07277 and YLL058W belonging to each pathway class.

Test sample	Likelihood for each pathway class	Remark a
C07277	*M*_1_: 1,123	Sum of confidence scores in row 1,3,6 of Table 2
	*M*_2_: 0	–
	*M*_3_: 0	–
	*M*_4_: 441	Sum of confidence scores in row 2 of Table 2
	*M*_5_: 0	–
	*M*_6_: 0	–
	*M*_7_: 0	–
	*M*_8_: 0	–
	*M*_9_: 918	Sum of confidence scores in row 1,4,5 of Table 2
	*M*_10_: 855	Sum of confidence scores in row 1,4 of Table 2
	*M*_11_: 0	–
YLL058W	*M*_1_: 900	Sum of confidence scores in row 8 of Table 2
	*M*_2_: 4,042	Sum of confidence scores in row 7,9,10,11,12,16,17 of Table 2
	*M*_3_: 0	–
	*M*_4_: 0	–
	*M*_5_: 4,621	Sum of confidence scores in row 8,9,10,12,13,16,17 of Table 2
	*M*_6_: 1,800	Sum of confidence scores in row 14,15 of Table 2
	*M*_7_: 0	–
	*M*_8_: 0	–
	*M*_9_: 0	–
	*M*_10_: 0	–
	*M*_11_: 0	–

a The information in this column shows the means by which the likelihood in column 2 was calculated by using the data in Table 2.

### Prediction Method

To describe the method more clearly, it is necessary to introduce some notations - let *M*_1_, *M*_2_, …, *M*_11_ denote 11 metabolic pathway classes, where *M*_1_ denotes “Carbohydrate Metabolism”, *M*_2_ the “Energy Metabolism”, and so forth (see column 1 and 2 of **Table 1**). In addition, if one supposes that there are *n* samples in the training set, say *s*_1_, *s*_2_, …, *s_n_*. The pathway class of a sample *s_i_* can be formulated as.
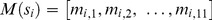
(1)where



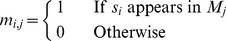
(2)Toward a query sample (small molecule or enzyme) *s*, its pathway class was predicted by not only its neighbors in the network but also the weights of edges between the query one and its neighbors. Let *N*(*s*) denote a node set consisting of the neighbors of *s*. The likelihood that *s* belongs to *M_j_* was calculated by.
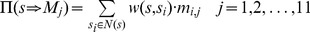
(3)where



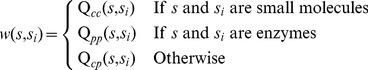
(4)Obviously, the larger the value of 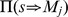 is, the more likely *s* belongs to *M_j_*. If 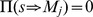 for some *j*, it implies that there are no interactive compounds or proteins of the query sample *s* in the training set that belong to pathway class *M_j_*. In this case, it is thought that the probability of *s* belonging to *M_j_* is zero. For a query sample *s*, if the results obtained from **Eq. 3** are
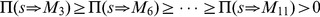
(5)which suggests that it is most likely that *s* belongs to is *M*_3_, followed by *M*_6_, and so forth. Also, *M*_3_ is called the 1-st order predicted pathway class of *s*, and *M*_6_ the 2-nd order predicted pathway class of *s*, and so forth.

**Table 4:** The prediction accuracies obtained by our method for small molecules, enzymes, and all samples.

Prediction order	Prediction accuracy forsmall molecules (S*_c_*)	Prediction accuracy forenzymes (S*_e_*)	Prediction accuracy fortotal samples S = S*_c_*∪S*_e_*
1	77.12%	92.05%	79.56%
2	19.12%	22.48%	19.67%
3	7.38%	10.55%	7.90%
4	3.61%	4.13%	3.70%
5	2.75%	4.13%	2.97%
6	1.40%	1.83%	1.47%
7	0.96%	0.76%	0.92%
8	0.51%	0.76%	0.55%
9	0.45%	0.61%	0.47%
10	0.30%	0.00%	0.25%
11	0.15%	0.00%	0.12%

### Jackknife Test

In statistical prediction, the jackknife test [19], one of the cross-validation methods, is often used to evaluate various predictors for their effectiveness. Compared with other cross-validation methods (independent dataset test and subsampling test), the jackknife test is deemed to be more objective [20], [21], [22]. For a given benchmark dataset, each sample can always be assigned to a unique predicted result through the jackknife test. Therefore, many investigators adopt this method to evaluate the accuracies of their predictors [19], [22], [23], [24], [25], [26], [27], [28], [29], [30], [31]. It was also adopted here to evaluate the generalization of predicting the metabolic pathways.

**Figure 3:**
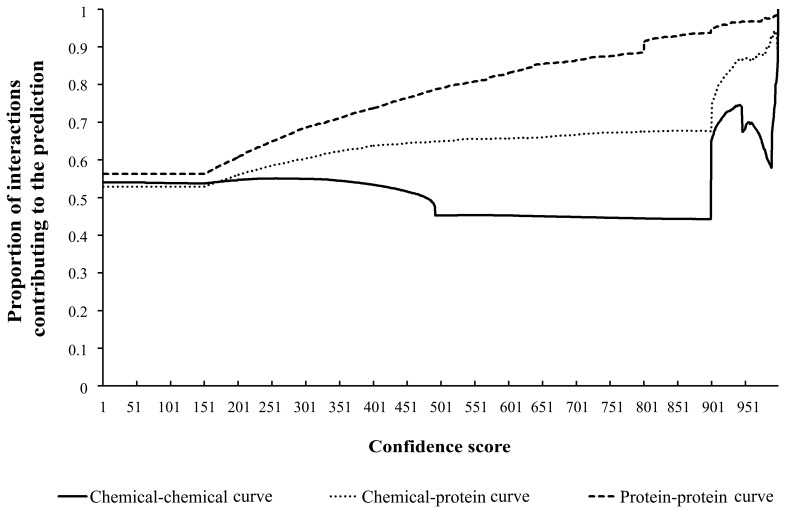
Three curves showing the changes of proportions of interactions contributing to the prediction when increasing the confidence score, where the chemical-chemical curve addresses chemical-chemical interactions, chemical-protein curve chemical-protein interactions, protein-protein curve protein-protein interactions. The X-axis is the confidence score. The Y-axis is the proportion of interactions contributing to the prediction. Generally, chemical-protein curve and protein-protein curve are ascending with the increase of confidence score, while chemical-chemical curve remains at a low level for low confidence scores and starts to increase quickly for high confidence scores.

### Accuracy Measurement

For any query sample (small molecule or enzyme), the prediction method described in Section “Prediction method” will provide a series of candidate pathway classes. For the *j-*th order predicted pathway class, its prediction accuracy 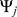
can be calculated by
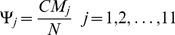
(6)where *CM_j_* denotes the number of samples that are predicted correctly according its *j*-th order predicted pathway class, and *N* denotes the total number of samples in the dataset. For these 11 prediction accuracies, high 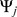 with small *j* and low 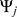
with large *j* indicate that the method arranges the candidate pathway classes well. The first order prediction accuracy is more important than others, because it has the smallest index of *j*.

Since the 11 prediction accuracies calculated by **Eq. 6** cannot evaluate the prediction method on the whole, another measurement is needed to calculate the probability of all pathway classes that are correctly predicted according to the first *m* predicted candidate pathways classes as follows [11], [15].
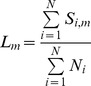
(7)where *S_i,m_* denotes the number of the correctly predicted pathway classes of the *i-*th sample among its first *m* predicted candidate pathway classes, and *N_i_* denotes the number of pathway classes that the *i-*th sample belongs to. Usually, we calculate **Eq. 7** by taking *m* as the smallest integer equal to or greater than the average number of samples’ pathway classes in the dataset, which is calculated by



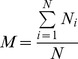
(8)Obviously, a large *L_m_* always implies a good performance for mapping small molecules or enzymes into correct metabolic pathway class.

**Table 5:** The distribution of samples with incorrect 1-st order predicted pathway class in 11 pathway classes.

Metabolic pathway class	Number of misclassified samples
Carbohydrate Metabolism	105
Energy Metabolism	32
Lipid Metabolism	79
Nucleotide Metabolism	26
Amino Acid Metabolism	146
Metabolism of Other Amino Acids	79
Glycan Biosynthesis and Metabolism	21
Metabolism of Cofactors and Vitamins	107
Metabolism of Terpenoids and Polyketides	107
Biosynthesis of Other Secondary Metabolites	95
Xenobiotics Biodegradation and Metabolism	113
Total	910 a

a The value in this cell is larger than the total number of samples with incorrect 1-st order prediction because some samples belong to more than one pathway class.

**Table 6:** Interactive compounds and enzymes of C00439 in pathway classes *M*_5_ and *M*_8._

Index	Interactive compounds and enzymes in *M*_5_		Interactive compounds and enzymes in *M*_8_	
	Compound/Enzyme	Confidence score	Compound/Enzyme	Confidence score
1	C01045	940	C00101	934
2	C00785	938	C00445	927
3	C00101	934	C00025	923
4	C00025	923	C00001	899
5	C00014	901	C00018	899
6	C03680	899	C00664	899
7	C01817	511	C03479	899
8	C05568	388	C14818	899
9	C00135	302	C14819	899
10	C00073	283	C00504	739
11	C02170	191	C00234	438
12	–	–	C00992	378
13	–	–	C00440	205
14	–	–	YGL125W	177
Likelihood	–	7,210	–	10,115

## Results and Discussion

### Performance of the Prediction Method for Small Molecules

In the training dataset, 3,348 small molecules comprised the dataset **S** *_c_*. The pathway classes of these molecules were predicted by the prediction method described in Section “Prediction method” by the jackknife test based on all samples in benchmark dataset. Here, an example is given to demonstrate how we made the prediction. “C07277”, belonging to *M*_9_, is a sample in **S** *_c_*. Its interactive compounds and proteins were shown from row 1 to 6 in **Table 2**. Using **Eq. 3**, the likelihood that “C07277” belongs to each of 11 pathway classes was calculated and shown in **Table 3**. As a result, “C07277” belongs to *M*_1_ with the highest likelihood, followed by *M*_9_, *M*_10_ and *M*_4_. The 1-st order predicted pathway class was not its true pathway class, while its 2-nd order predicted pathway class was its true pathway class. After the pathway classes of each sample in **S** *_c_* were predicted, 11 ordered prediction accuracies were obtained by **Eq. 6** and listed in column 2 of **Table 4**, from which we can see that the first order prediction accuracy was 77.12%. It is also observed form column 2 of **Table 4** that the prediction accuracy generally followed a descending trend when increasing the order number, which indicates that our method sorted the predicted pathway classes well. The average number of pathway classes for small molecules was 1.15 (3,844/3,348) according to **Eq. 8**, *i.e.*, *M* = 1.15. Thus we consider the first 2 predicted pathway classes for each small molecule. After collecting these pathway classes calculated according to **Eq. 7**, it was observed that the probability that all true pathway classes were covered by them was 83.81%. Our results are comparable to that in [8], where the results were obtained by only chemical-chemical interactions.

### Performance of the Prediction Method for Enzymes

In addition to the small molecules, there were 654 enzymes in the training dataset, which comprised dataset **S** *_e_*. Our prediction method was also applied to predict their metabolic pathway classes, evaluated by the jackknife test. Likewise, “YLL058W”, a sample in **S** *_e_*, was selected to demonstrate how its predicted pathway classes were obtained. “YLL058W” belongs to two pathway classes: *M*_2_ and *M*_5_. Its interactive compounds and proteins were shown from row 7 to 17 in **Table 2** and the likelihood of “YLL058W” belonging to each of 11 pathway classes was shown in **Table 3**, from which we can see that “YLL058W” belonging to *M*_5_ is most likely, followed by *M*_2_, *M*_6_ and *M*_1_. The first two predicted pathway classes were its true pathway classes. After processing by **Eq. 6**, 11 ordered prediction accuracies were obtained. These accuracies were listed in column 3 of **Table 4**, from which we can see that the first order prediction accuracy was 92.05%. The average number of pathway classes for enzymes was 1.37 (898/654) according to **Eq. 8**, *i.e.*, *M* = 1.37, meaning that the average success rate by a random guess would be 12.46% (1.37/11), which is much lower than that by our method. Like the 11 ordered prediction accuracies for small molecules, those for enzymes also generally followed a descending trend when increasing the order number (cf. **Table 4**), which suggests that the predicted pathway classes were sorted quite well. As described above, the average number of pathway classes for enzymes was 1.37. **Eq. 7** was calculated by taking *m* = 2, yielding a probability of 83.41% that all true pathway classes were covered by the first 2 predicted classes.

### Performance of the Prediction Method for All Samples

The predicted results for all samples in the benchmark dataset **S** combined the results of small molecules in dataset **S** *_c_* and enzymes in dataset **S** *_e_*. Listed in column 4 of **Table 4** were 11 ordered prediction accuracies, from which the first ordered prediction accuracy was 79.56%. The average number of pathway classes for the samples in **S** was 1.18 (4,742/4,002) according to **Eq. 8**, *i.e.*, *M* = 1.18, meaning that the average success rate by a random guess would be 10.73% (1.18/11), much lower than that obtained by our method. Meanwhile, it is observed from column 4 of **Table 4** that the 11 prediction accuracies followed a descending trend when increasing the order number, suggesting that the predicted pathway classes, for both small molecules and enzymes, were sorted quite well by our method. Since the average number of pathway classes for all samples in **S** was 1.18, the first two predicted pathway classes for each sample were considered. After collecting these pathway classes calculated by **Eq. 7** by taking *m* = 2, 83.74% true pathway classes were covered by the first 2 predicted pathway classes.

### Confidence Scores of Small Molecules or Enzymes

As illustrated by the above sections, our method is very effective in predicting the metabolic pathway classes of small molecules and enzymes, indicating that interactive small molecules or enzymes are very likely to appear in a common metabolic pathway. In this section, we analyze the confidence score and illustrate the value in utilizing such scores.

The network constructed contains 4,002 samples and 100,754 interactions, including 66,942 chemical-chemical interactions, 19,695 chemical-protein interactions, and 14,117 protein-protein interactions. As described in Section “Construction of hybrid network”, each interaction was labeled with a confidence score ranging from 1 to 999, quantifying the likelihood that an interaction occurs. For each integer *k* in the interval [1, 999], the following rate was calculated for each kind of interaction.
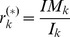
(9)where *I_k_* is the number of interactions with confidence score to be at least *k*, and *IM_k_* is the number of interactions with their confidence score to be at least *k* and their corresponding small molecules or enzymes belonging to at least one common pathway class. The superscript of **Eq. 9** was to differentiate three different kinds of interactions – 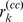
is for chemical-chemical interaction, 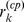 for chemical-protein interaction, and 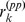 for protein-protein interaction. It is clear that the value of **Eq. 9** quantifies the contribution of the interactions with confidence score at least *k* for predicting the pathway classes of small molecules and enzymes in our method. For each kind of interaction, we can plot a curve with 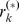
as its Y-axis and the subscript *k* as its X-axis. For clarity, the curve for chemical-chemical interactions is named the chemical-chemical curve, the curve for chemical-protein interactions is the chemical-protein curve, and that for protein-protein interactions is the protein-protein curve. Shown in **Figure 3** are three curves, from which we can see that the chemical-protein curve and protein-protein curve generally follow an increasing trend when increasing the confidence score; while the chemical-chemical curve does not look good in terms of its overall trend – the rate remains at a low level (between 40%−60%) when *k* < ∼900, and when k > ∼900, the rate starts to increase quickly. These data indicate that the proportions of the interactions contributing to the prediction in the method become higher and higher with the increasing of confidence score, meaning that the confidence scores of interactions are related to the prediction of enzymes and compounds in a metabolic pathway. It is, therefore, foreseeable that as the interactions become more evidenced in STRING and STITCH, predictions requiring confidence scores will also be improved accordingly. Finally, it is important to note that when taking all interactive enzymes or compounds into consideration, more than half of the interactions would provide contributions to the prediction, indicating that using interaction information of proteins and chemicals to predict their metabolic pathways is reasonable. It is also the basis upon which our method performs well.

### Analysis of Samples with Incorrect 1-st Order Predictions

Although our method performs well, where the 1-st order prediction accuracy for all samples achieved 79.56%, 818 samples (818/4002, 20.44%) achieved incorrect 1-st order predictions. The distribution of these misclassified samples in the 11 pathway classes is shown in **Table 5**. We investigate these samples in depth and explain why these samples were misclassified as follows. Based on the principle of the method, the likelihood that a misclassified sample belongs to its 1-st order predicted pathway class was greater than those of true pathway classes, while the likelihood of a sample belonging to one class is calculated by summing the confidence scores between the sample and its neighbors belonging to that class. Thus, it would be interesting to investigate sum terms of the likelihood that a misclassified sample belongs to a 1-st order predicted pathway class and true pathway classes. The misclassified sample “C00439” belongs to pathway class *M*_5_, while its 1-st order predicted pathway class was *M*_8_. Shown in **Table 6** are the interactive compounds and enzymes of “C00439” in *M*_5_ and *M*_8_, and the last row of **Table 6** shows the likelihood of “C00439” belonging to *M*_5_ and *M*_8_. Two difficult situations were observed from **Table 6** as follows: (1) sum terms for 1-st order predicted pathway class were greater than those of true pathway classes; (2) sum terms with values greater than 700, which is deemed the threshold of interactions with high confidence [32], [33], for 1-st order predicted pathway class were greater than those of true pathway classes. Due to the method of calculating the likelihood (cf. **Eq. 3**), it is highly possible that a query sample satisfying one of the above situations would be predicted incorrectly. Among 818 misclassified samples, 556 (556/818, 67.97%) samples fit the first situation; while 604 (604/818, 73.84%) samples fit the second situation. Furthermore, 762 (762/818, 93.15%) samples fit at least one of the two situations. As a result, these samples were all misclassified. On the other hand, the incompleteness of the interaction information may be another important reason. When interactions, especially those with high confidence scores, for the true class are missing in the calculation, the prediction is likely to be incorrect.

### Conclusions

By integrating the data for chemical-chemical interactions, chemical-protein interactions, and protein-protein interactions, a multi-label prediction model was developed to identify the metabolic pathway classes of small molecules and enzymes. Since interactive chemicals and proteins are more likely to involve a common pathway, the first order prediction accuracy achieved by our method was 79.56%, much higher than the average success rate by a random guess. Our analysis shows that interactive chemicals or proteins with higher confidence scores would be more likely to participate in the same metabolic pathway. We hope that this method may facilitate the understanding of metabolic pathway systems. It is also anticipated that prediction accuracy will increase as more and more interaction information concerning chemicals and proteins becomes available.

## Supporting Information

Online Supporting Information S1 **List of the 4,002 samples, including 3,348 small molecules and 654 enzymes of yeast, classified into 11 metabolic pathway classes.**
(PDF)
