# Correction: Predicting Metabolic Pathways of Small Molecules and Enzymes Based on Interaction Information of Chemicals and Proteins

**DOI:** 10.1371/annotation/83922541-168a-4d4f-846a-cb5d127aa7a9

**Published:** 2012-11-02

**Authors:** Yu-Fei Gao, Lei Chen, Yu-Dong Cai, Kai-Yan Feng, Tao Huang, Yang Jiang

There were errors in the Funding section. The correct funding information is as follows: This contribution is supported by National Basic Research Program of China(2011CB510102, 2011CB510101), Innovation Program of Shanghai Municipal

Education Commission (No.12YZ120, No. 12ZZ087), Natural science fund projects of Jilin province (201215059), development of science and technology plan projects of Jilin province (20100733, 201101074), SRF for ROCS, SEM (2009-36), Scientific Research Foundation (Jilin Department of Science #xamp; Technology, 200705314, 20090175, 20100733), Scientific Research Foundation (Jilin Department of Health, 2010Z068), SRF for ROCS (Jilin Department of Human Resource #xamp; Social Security, 2012-2014). The funders had no role in study design, data collection and analysis, decision to publish, or preparation of the manuscript. 

