# Supplementary material for: Predicting Metabolic Pathways of Small Molecules and Enzymes Based on Interaction Information of Chemicals and Proteins
Source: PLoS One. 2012 Sep 21;7(9):e45944. doi: 10.1371/journal.pone.0045944 (PMC3448724; doi:10.1371/journal.pone.0045944)
Supplement: Online Supporting Information S1 — List of the 4,002 samples, including 3,348 small molecules and 654 enzymes of yeast, classified into 11 metabolic pathway classes. (PDF) [file pone.0045944.s001.pdf]

Online Supporting Information S1. The benchmark dataset **S** contains 4,002 samples, with 3,348 small molecules and 654 enzymes of yeast. These samples are classified into 11 metabolic pathway classes.

1. 592 samples in “Carbohydrate Metabolism”

(I) 394 small molecules

C00011	C00022	C00024	C00025	C00026	C00029	C00031	C00033
C00036	C00042	C00043	C00048	C00052	C00058	C00068	C00072
C00074	C00083	C00084	C00085	C00089	C00091	C00092	C00095
C00096	C00099	C00100	C00103	C00109	C00111	C00116	C00117
C00118	C00119	C00121	C00122	C00124	C00128	C00136	C00137
C00140	C00149	C00158	C00159	C00160	C00163	C00164	C00167
C00168	C00181	C00183	C00185	C00186	C00190	C00191	C00197
C00198	C00199	C00203	C00204	C00207	C00208	C00209	C00221
C00222	C00227	C00231	C00232	C00236	C00243	C00246	C00247
C00252	C00256	C00257	C00258	C00259	C00266	C00267	C00270
C00275	C00279	C00309	C00310	C00311	C00312	C00313	C00325
C00329	C00332	C00333	C00334	C00345	C00352	C00356	C00357
C00379	C00392	C00394	C00417	C00424	C00433	C00446	C00469
C00470	C00474	C00476	C00490	C00492	C00497	C00498	C00501
C00502	C00507	C00508	C00514	C00531	C00532	C00545	C00546
C00552	C00558	C00577	C00583	C00617	C00618	C00620	C00631
C00636	C00644	C00645	C00651	C00663	C00665	C00668	C00672
C00673	C00679	C00683	C00684	C00689	C00691	C00734	C00741
C00789	C00794	C00798	C00800	C00804	C00810	C00817	C00818
C00827	C00861	C00877	C00879	C00880	C00888	C00894	C00898
C00900	C00905	C00935	C00937	C00973	C00975	C00976	C00988
C00989	C01011	C01013	C01019	C01040	C01041	C01050	C01068
C01083	C01089	C01094	C01096	C01097	C01099	C01101	C01109
C01113	C01114	C01115	C01127	C01131	C01132	C01144	C01146
C01151	C01159	C01170	C01172	C01177	C01182	C01204	C01213
C01216	C01218	C01219	C01220	C01222	C01231	C01234	C01235
C01236	C01243	C01245	C01251	C01272	C01284	C01286	C01380
C01384	C01412	C01451	C01508	C01613	C01620	C01623	C01674
C01680	C01697	C01721	C01732	C01769	C01788	C01801	C01825
C01845	C01904	C01934	C01989	C01990	C02013	C02123	C02170
C02199	C02225	C02226	C02262	C02266	C02273	C02280	C02330
C02331	C02335	C02336	C02405	C02411	C02431	C02504	C02527
C02591	C02612	C02614	C02630	C02670	C02713	C02876	C02888
C02977	C02985	C02995	C03044	C03046	C03058	C03117	C03217
C03248	C03267	C03289	C03291	C03383	C03410	C03451	C03459
C03546	C03548	C03561	C03598	C03599	C03618	C03659	C03660
C03737	C03752	C03785	C03826	C03906	C03921	C03979	C03981
C04006	C04037	C04053	C04062	C04063	C04089	C04225	C04257

C04287	C04297	C04348	C04349	C04442	C04477	C04501	C04520
C04546	C04563	C04573	C04575	C04579	C04593	C04631	C05125
C05345	C05378	C05379	C05382	C05385	C05396	C05399	C05400
C05401	C05402	C05404	C05411	C05412	C05422	C05668	C05731
C05984	C05985	C05986	C05993	C05999	C06002	C06010	C06019
C06023	C06027	C06028	C06029	C06030	C06032	C06033	C06118
C06142	C06146	C06156	C06186	C06187	C06188	C06192	C06215
C06216	C06217	C06218	C06219	C06240	C06241	C06311	C06376
C06377	C06441	C06473	C06892	C06893	C11516	C11521	C11544
C11555	C13952	C14899	C15923	C15925	C15926	C15930	C16153
C16154	C16155	C16156	C16186	C16688	C16698	C17327	C17328
C18026	C18324						

(II) 198 enzymes

YAL038W	YAL054C	YAL060W	YAL061W	YBL015W
YBL068W	YBR001C	YBR006W	YBR018C	YBR019C
YBR020W	YBR023C	YBR038W	YBR084W	YBR117C
YBR126C	YBR145W	YBR196C	YBR218C	YBR221C
YBR299W	YCL009C	YCL040W	YCR005C	YCR012W
YCR036W	YDL021W	YDL037C	YDL055C	YDL066W
YDL078C	YDL080C	YDL103C	YDL131W	YDL168W
YDL174C	YDL182W	YDL246C	YDR001C	YDR009W
YDR050C	YDR074W	YDR148C	YDR173C	YDR178W
YDR208W	YDR248C	YDR261C	YDR272W	YDR287W
YEL011W	YEL058W	YEL070W	YER003C	YER065C
YER073W	YER099C	YER178W	YFL017C	YFL018C
YFL045C	YFR015C	YFR019W	YFR053C	YGL062W
YGL253W	YGL256W	YGR019W	YGR032W	YGR043C
YGR087C	YGR192C	YGR194C	YGR204W	YGR240C
YGR244C	YGR248W	YGR254W	YGR256W	YGR282C
YGR287C	YGR292W	YHL011C	YHL012W	YHR046C
YHR104W	YHR163W	YHR174W	YHR183W	YIL043C
YIL099W	YIL107C	YIL125W	YIL162W	YIL172C
YIR019C	YIR031C	YJL045W	YJL052W	YJL121C
YJL153C	YJL155C	YJL200C	YJL216C	YJL221C
YJR009C	YJR153W	YJR159W	YKL029C	YKL035W
YKL060C	YKL085W	YKL104C	YKL127W	YKL141W
YKL148C	YKL150W	YKL152C	YKL181W	YKR043C
YKR097W	YLL041C	YLR044C	YLR070C	YLR153C
YLR164W	YLR174W	YLR240W	YLR258W	YLR286C
YLR300W	YLR304C	YLR305C	YLR307W	YLR308W
YLR342W	YLR354C	YLR377C	YML004C	YML054C
YML100W	YML126C	YMR083W	YMR099C	YMR105C
YMR108W	YMR118C	YMR169C	YMR170C	YMR205C
YMR250W	YMR261C	YMR278W	YMR303C	YMR306W

YMR323W YNL009W YNL037C YNL071W YNL104C
YNL106C YNL117W YNL192W YNL241C YNL267W
YNR001C YNR016C YNR034W YOL061W YOL065C
YOL086C YOL126C YOL136C YOL157C YOR040W
YOR095C YOR109W YOR136W YOR142W YOR190W
YOR347C YOR374W YOR388C YOR393W YPL017C
YPL028W YPL061W YPL262W YPL268W YPR001W
YPR002W YPR006C YPR026W YPR074C YPR113W
YPR160W YPR184W YCR073W-A

2. 297 samples in “Energy Metabolism”

(I) 151 small molecules

C00001 C00002 C00003 C00004 C00005 C00006 C00007 C00008
C00009 C00011 C00013 C00014 C00022 C00024 C00025 C00026
C00033 C00034 C00036 C00037 C00041 C00042 C00048 C00049
C00053 C00054 C00058 C00059 C00061 C00064 C00065 C00067
C00074 C00080 C00083 C00085 C00087 C00088 C00091 C00094
C00097 C00100 C00101 C00111 C00117 C00118 C00122 C00132
C00143 C00149 C00152 C00155 C00158 C00168 C00169 C00184
C00192 C00197 C00199 C00218 C00222 C00224 C00227 C00231
C00232 C00234 C00236 C00237 C00244 C00258 C00263 C00279
C00282 C00283 C00288 C00311 C00320 C00332 C00354 C00390
C00399 C00417 C00440 C00445 C00447 C00488 C00533 C00536
C00542 C00543 C00565 C00593 C00631 C00683 C00697 C00862
C00876 C00877 C00887 C00894 C00979 C00989 C01001 C01005
C01011 C01013 C01031 C01046 C01077 C01080 C01104 C01118
C01144 C01179 C01182 C01213 C01217 C01274 C01330 C01353
C01417 C01438 C01563 C01861 C02061 C02076 C02185 C02331
C03232 C03576 C03920 C04330 C04348 C04377 C04488 C04628
C04732 C04832 C05382 C05528 C05668 C06019 C06027 C06028
C11062 C11536 C11537 C11903 C11904 C14180 C18323

(II) 146 enzymes

Q0045 Q0065 Q0070 Q0080 Q0085
Q0105 Q0110 Q0115 Q0120 Q0130
Q0250 Q0275 YAL012W YAL038W YAL054C
YAL062W YBL045C YBL099W YBR011C YBR039W
YBR117C YBR127C YBR263W YCL050C YCR012W
YDL004W YDL021W YDL067C YDL078C YDL085W
YDL168W YDL171C YDL185W YDL215C YDR019C
YDR050C YDR111C YDR178W YDR256C YDR298C
YDR321W YDR376W YDR377W YDR529C YEL024W
YEL027W YEL051W YER081W YFL053W YFR030W
YFR033C YFR055W YGL008C YGL125W YGL184C
YGL187C YGL191W YGR012W YGR020C YGR088W

YGR124W	YGR183C	YGR208W	YGR240C	YGR254W
YHR026W	YHR051W	YHR174W	YIL074C	YIL111W
YJL045W	YJL068C	YJL121C	YJL166W	YJR010W
YJR121W	YJR130C	YJR137C	YKL001C	YKL016C
YKL060C	YKL080W	YKL085W	YKL106W	YKL141W
YKL148C	YKL152C	YKL192C	YKR043C	YKR097W
YLL041C	YLL058W	YLR027C	YLR038C	YLR058C
YLR089C	YLR153C	YLR155C	YLR157C	YLR158C
YLR160C	YLR164W	YLR295C	YLR303W	YLR377C
YLR395C	YLR447C	YML070W	YML082W	YML120C
YMR054W	YMR118C	YMR145C	YMR205C	YMR256C
YMR267W	YMR323W	YNL052W	YNL277W	YOL064C
YOL126C	YOR065W	YOR095C	YOR184W	YOR270C
YOR332W	YOR347C	YOR375C	YOR388C	YOR393W
YPL023C	YPL036W	YPL078C	YPL234C	YPL271W
YPR020W	YPR035W	YPR036W	YPR074C	YPR145W
YPR167C	YPR191W	YHR001W-A	YHR039C-A	YML081C-A
YOL077W-A				

3. 483 samples in “Lipid Metabolism”

(I) 399 small molecules

C00010	C00024	C00029	C00037	C00065	C00083	C00084	C00093
C00100	C00111	C00114	C00116	C00136	C00154	C00164	C00184
C00187	C00189	C00197	C00207	C00219	C00245	C00249	C00258
C00280	C00307	C00319	C00332	C00346	C00356	C00410	C00412
C00427	C00448	C00468	C00479	C00489	C00510	C00513	C00517
C00523	C00527	C00535	C00570	C00577	C00583	C00584	C00588
C00599	C00639	C00670	C00674	C00695	C00696	C00712	C00735
C00751	C00762	C00823	C00836	C00877	C00909	C00951	C00969
C01054	C01089	C01120	C01124	C01144	C01164	C01176	C01189
C01210	C01226	C01227	C01233	C01301	C01312	C01530	C01561
C01571	C01595	C01673	C01694	C01724	C01747	C01753	C01780
C01789	C01794	C01802	C01832	C01902	C01921	C01943	C01944
C01953	C01996	C02041	C02050	C02140	C02141	C02165	C02166
C02198	C02249	C02373	C02457	C02528	C02537	C02538	C02593
C02679	C02934	C02990	C03035	C03205	C03242	C03428	C03547
C03561	C03577	C03594	C03595	C03640	C03681	C03772	C03845
C03852	C03872	C03917	C03990	C04042	C04056	C04295	C04373
C04483	C04518	C04525	C04554	C04555	C04577	C04672	C04676
C04717	C04722	C04742	C04780	C04785	C04805	C04822	C04853
C05103	C05107	C05108	C05109	C05122	C05138	C05139	C05140
C05141	C05259	C05261	C05264	C05267	C05269	C05270	C05272
C05274	C05275	C05284	C05285	C05290	C05291	C05293	C05294
C05295	C05296	C05297	C05298	C05299	C05300	C05301	C05302

C05337	C05356	C05401	C05437	C05439	C05440	C05441	C05442
C05443	C05444	C05445	C05446	C05447	C05448	C05449	C05450
C05451	C05452	C05453	C05454	C05455	C05460	C05465	C05466
C05467	C05469	C05470	C05471	C05472	C05476	C05479	C05480
C05481	C05482	C05484	C05485	C05488	C05490	C05497	C05498
C05500	C05501	C05502	C05503	C05504	C05949	C05950	C05951
C05952	C05953	C05954	C05956	C05957	C05958	C05959	C05960
C05961	C05962	C05963	C05964	C05965	C05966	C05979	C05999
C06083	C06124	C06309	C06310	C06314	C06341	C06423	C06424
C06425	C06426	C06427	C06428	C06429	C06462	C06771	C06772
C07289	C07354	C07880	C08281	C08316	C08320	C08323	C08357
C08362	C08491	C08821	C08830	C11133	C11134	C11135	C11455
C11508	C11512	C11521	C11522	C11523	C12144	C13482	C13550
C13712	C13713	C13809	C14717	C14732	C14748	C14749	C14762
C14765	C14766	C14767	C14772	C14773	C14774	C14775	C14776
C14777	C14778	C14780	C14781	C14782	C14807	C14808	C14810
C14811	C14812	C14813	C14814	C14815	C14822	C14823	C14824
C14826	C14827	C14828	C14829	C14831	C14834	C14835	C15515
C15517	C15518	C15519	C15520	C15610	C15613	C15776	C15777
C15778	C15780	C15781	C15782	C15783	C15808	C15816	C15915
C16162	C16300	C16308	C16309	C16310	C16311	C16316	C16317
C16319	C16320	C16322	C16331	C16335	C16344	C16345	C16513
C16522	C16525	C16526	C16527	C16528	C16529	C16530	C16532
C17332	C17333	C17337	C17339	C17343	C17345	C17346	C17644
C17647	C17649	C17650	C17654	C17657	C17658	C17660	C17661
C17662	C17664	C17667	C17687	C17726	C17727	C17737	C18038
C18040	C18041	C18042	C18043	C18044	C18045	C18075	

(II) 84 enzymes

YBL011W	YBR026C	YBR029C	YBR145W	YBR244W
YBR265W	YCL004W	YCR048W	YDL022W	YDL052C
YDL168W	YDR058C	YDR062W	YDR147W	YDR294C
YDR402C	YER015W	YER026C	YER061C	YER062C
YER073W	YFL053W	YGL001C	YGL012W	YGL055W
YGL205W	YGL256W	YGR007W	YGR060W	YGR157W
YGR170W	YGR175C	YGR202C	YHL003C	YHL032C
YHR007C	YHR072W	YHR104W	YHR123W	YHR190W
YIL009W	YIL049W	YIL053W	YIL124W	YIL160C
YIR037W	YJR019C	YKL008C	YKL026C	YKL182W
YKR031C	YKR067W	YLR100W	YLR133W	YLR260W
YLR299W	YML008C	YML070W	YML126C	YMR006C
YMR008C	YMR083W	YMR246W	YMR296C	YMR303C
YNL045W	YNL130C	YNL169C	YNL280C	YNR008W
YNR016C	YNR019W	YOL011W	YOL059W	YOL086C
YOR171C	YOR175C	YOR317W	YOR374W	YPL028W

YPL061W YPL206C YPL231W YPR113W

4. 240 samples in "Nucleotide Metabolism"

(I) 133 small molecules

C00002 C00008 C00011 C00014 C00015 C00020 C00029 C00035
C00037 C00044 C00048 C00053 C00054 C00055 C00059 C00063
C00064 C00075 C00081 C00086 C00099 C00104 C00105 C00106
C00112 C00117 C00119 C00130 C00131 C00144 C00147 C00169
C00178 C00206 C00212 C00214 C00224 C00239 C00242 C00262
C00286 C00294 C00295 C00299 C00301 C00330 C00337 C00360
C00361 C00362 C00363 C00364 C00365 C00366 C00380 C00383
C00385 C00387 C00429 C00438 C00458 C00459 C00460 C00475
C00499 C00526 C00559 C00575 C00603 C00620 C00655 C00672
C00700 C00705 C00802 C00813 C00881 C00906 C00942 C01103
C01168 C01228 C01260 C01261 C01345 C01346 C01367 C01368
C01762 C02067 C02091 C02170 C02348 C02350 C02353 C02354
C02355 C02376 C02642 C02718 C03090 C03373 C03483 C03794
C03838 C03997 C04051 C04376 C04494 C04640 C04677 C04734
C04751 C04823 C05100 C05145 C05239 C05512 C05513 C05515
C05516 C05822 C05993 C06193 C06194 C06195 C06196 C06197
C06198 C11821 C12248 C15607 C15667

(II) 107 enzymes

YAL038W YAR015W YBL039C YBL068W YBR111C
YBR252W YBR278W YCL050C YCR014C YDL102W
YDL140C YDL150W YDL238C YDR045C YDR121W
YDR156W YDR226W YDR353W YDR408C YDR419W
YDR441C YDR454C YDR530C YEL021W YEL055C
YER005W YER070W YER099C YER170W YFL036W
YGL063W YGL234W YGL248W YGR061C YGR180C
YHL011C YHR106W YHR128W YHR144C YHR201C
YHR216W YIL066C YIL139C YIR027C YIR029W
YIR032C YJL005W YJL026W YJL130C YJL148W
YJR010W YJR057W YJR063W YJR103W YJR105W
YJR109C YKL001C YKL024C YKL067W YKL127W
YKL144C YKL181W YKL216W YLR028C YLR209C
YLR245C YLR359W YLR420W YLR432W YML022W
YML035C YML056C YML106W YMR105C YMR120C
YMR217W YMR271C YMR278W YMR300C YNL102W
YNL113W YNL141W YNL151C YNL220W YNL248C
YNL262W YNR003C YNR012W YOL061W YOL115W
YOR074C YOR116C YOR128C YOR151C YOR207C
YOR303W YOR330C YOR340C YOR341W YOR347C
YOR360C YPL167C YPR010C YPR062W YPR110C
YPR175W YPR190C

5. 647 samples in “Amino Acid Metabolism”

(I) 489 small molecules

C00011	C00014	C00019	C00021	C00022	C00024	C00025	C00026
C00036	C00037	C00041	C00042	C00047	C00048	C00049	C00051
C00059	C00062	C00064	C00065	C00068	C00073	C00074	C00077
C00078	C00079	C00082	C00083	C00084	C00086	C00091	C00094
C00097	C00100	C00101	C00108	C00109	C00114	C00119	C00122
C00123	C00134	C00135	C00141	C00143	C00146	C00148	C00152
C00155	C00156	C00164	C00166	C00168	C00169	C00170	C00179
C00180	C00183	C00188	C00197	C00213	C00223	C00230	C00232
C00233	C00251	C00254	C00258	C00263	C00279	C00283	C00296
C00300	C00315	C00320	C00322	C00323	C00327	C00328	C00331
C00332	C00334	C00352	C00355	C00356	C00388	C00398	C00402
C00406	C00407	C00408	C00409	C00423	C00430	C00431	C00433
C00436	C00437	C00438	C00439	C00441	C00449	C00450	C00463
C00483	C00487	C00489	C00491	C00493	C00506	C00512	C00527
C00530	C00533	C00544	C00546	C00547	C00555	C00576	C00581
C00582	C00596	C00601	C00606	C00624	C00628	C00630	C00632
C00637	C00642	C00643	C00666	C00671	C00680	C00683	C00719
C00739	C00740	C00750	C00755	C00763	C00780	C00785	C00788
C00791	C00793	C00805	C00811	C00822	C00826	C00860	C00877
C00884	C00940	C00944	C00954	C00955	C00956	C00957	C00978
C00979	C00986	C00990	C01005	C01010	C01026	C01028	C01035
C01036	C01042	C01043	C01044	C01045	C01060	C01061	C01077
C01100	C01102	C01110	C01118	C01137	C01142	C01144	C01149
C01152	C01157	C01161	C01165	C01179	C01180	C01181	C01186
C01198	C01207	C01213	C01234	C01250	C01251	C01252	C01259
C01262	C01267	C01269	C01302	C01575	C01586	C01598	C01672
C01682	C01693	C01717	C01772	C01817	C01829	C01850	C01877
C01888	C01929	C01962	C01987	C02043	C02137	C02161	C02167
C02170	C02172	C02218	C02220	C02226	C02265	C02291	C02298
C02305	C02362	C02442	C02465	C02470	C02504	C02505	C02514
C02515	C02565	C02612	C02631	C02637	C02647	C02693	C02700
C02714	C02727	C02739	C02741	C02763	C02765	C02775	C02835
C02937	C02938	C02939	C02946	C02989	C03063	C03069	C03077
C03078	C03082	C03087	C03089	C03090	C03145	C03166	C03175
C03194	C03227	C03230	C03231	C03232	C03239	C03273	C03277
C03283	C03284	C03287	C03296	C03340	C03344	C03345	C03375
C03406	C03415	C03440	C03453	C03460	C03506	C03508	C03519
C03539	C03564	C03589	C03656	C03672	C03680	C03722	C03758
C03765	C03771	C03793	C03794	C03824	C03871	C03912	C03955
C03972	C04002	C04020	C04043	C04044	C04045	C04052	C04076
C04092	C04133	C04137	C04148	C04185	C04186	C04188	C04236
C04272	C04281	C04282	C04302	C04368	C04390	C04405	C04409

C04411 C04421 C04462 C04479 C04498 C04582 C04642 C04666
C04677 C04691 C04877 C04882 C04896 C04916 C05125 C05127
C05130 C05135 C05161 C05235 C05332 C05338 C05519 C05527
C05528 C05539 C05548 C05554 C05565 C05568 C05570 C05572
C05575 C05576 C05577 C05578 C05579 C05580 C05581 C05582
C05583 C05584 C05585 C05587 C05588 C05589 C05593 C05594
C05598 C05600 C05604 C05607 C05620 C05629 C05634 C05635
C05637 C05638 C05639 C05640 C05642 C05643 C05645 C05646
C05651 C05653 C05658 C05659 C05660 C05662 C05663 C05823
C05824 C05827 C05828 C05829 C05830 C05831 C05834 C05837
C05852 C05853 C05931 C05932 C05933 C05936 C05939 C05942
C05945 C05946 C05947 C06000 C06001 C06002 C06006 C06007
C06010 C06032 C06044 C06046 C06047 C06048 C06181 C06199
C06212 C06213 C06231 C06442 C06547 C06866 C07086 C07303
C08276 C08300 C08301 C09306 C10447 C10497 C11457 C11481
C11588 C12621 C12623 C12986 C12987 C12988 C12989 C15524
C15532 C15606 C15650 C15651 C15699 C15700 C15767 C15980
C16069 C16432 C16666 C16673 C16719 C16848 C16850 C17203
C17268 C17935 C17938 C18048 C18172 C18174 C18202 C18325
C18326

(II) 158 enzymes

YAL012W YAL062W YBL076C YBL098W YBR006W
YBR115C YBR145W YBR166C YBR208C YBR221C
YBR249C YBR263W YCL009C YCL018W YCL030C
YCL064C YCR053W YDL131W YDL168W YDL171C
YDL182W YDL215C YDR007W YDR019C YDR035W
YDR111C YDR127W YDR148C YDR158W YDR232W
YDR234W YDR242W YDR256C YDR268W YDR300C
YDR321W YDR354W YDR402C YDR440W YDR502C
YEL038W YEL046C YER023W YER026C YER043C
YER052C YER055C YER069W YER073W YER081W
YER086W YER090W YER091C YER178W YFL018C
YFR025C YFR055W YGL009C YGL026C YGL148W
YGL184C YGL202W YGL256W YGR012W YGR019W
YGR088W YGR094W YGR124W YGR155W YGR208W
YHR018C YHR025W YHR037W YHR119W YHR137W
YHR208W YIL020C YIL074C YIL094C YIL116W
YIL125W YIL160C YIR034C YJL088W YJL130C
YJL168C YJR016C YJR024C YJR025C YJR078W
YJR109C YJR130C YJR139C YJR148W YKL104C
YKL106W YKL184W YKL211C YLL058W YLR017W
YLR027C YLR058C YLR089C YLR134W YLR142W
YLR146C YLR155C YLR157C YLR158C YLR160C
YLR180W YLR231C YLR303W YLR355C YLR359W

YLR382C	YLR438W	YML082W	YML126C	YMR009W
YMR062C	YMR083W	YMR108W	YMR169C	YMR170C
YMR189W	YMR250W	YMR300C	YMR303C	YNL104C
YNL220W	YNL277W	YNL316C	YNR050C	YOL052C
YOL058W	YOL086C	YOL097C	YOL140W	YOR184W
YOR202W	YOR251C	YOR303W	YOR323C	YOR374W
YOR375C	YPL017C	YPL028W	YPL040C	YPL061W
YPL111W	YPL160W	YPR035W	YPR060C	YPR062W
YPR069C	YPR118W	YPR145W		

6. 200 samples in “Metabolism of Other Amino Acids”

(I) 156 small molecules

C00005	C00006	C00011	C00014	C00022	C00024	C00025	C00026
C00033	C00037	C00041	C00049	C00051	C00062	C00064	C00065
C00072	C00074	C00077	C00082	C00083	C00084	C00094	C00097
C00099	C00100	C00106	C00127	C00133	C00134	C00135	C00152
C00177	C00189	C00217	C00222	C00227	C00245	C00302	C00315
C00334	C00383	C00429	C00488	C00506	C00515	C00519	C00555
C00593	C00606	C00650	C00669	C00692	C00736	C00750	C00792
C00804	C00819	C00864	C00894	C00986	C00993	C01013	C01050
C01073	C01110	C01212	C01262	C01326	C01401	C01419	C01528
C01594	C01672	C01678	C01855	C01879	C01959	C02090	C02237
C02335	C02512	C02535	C02642	C02659	C02798	C03149	C03167
C03170	C03341	C03557	C03564	C03646	C03722	C03742	C03771
C03943	C04353	C05042	C05122	C05123	C05143	C05172	C05335
C05341	C05422	C05665	C05668	C05669	C05670	C05672	C05679
C05680	C05682	C05684	C05686	C05688	C05689	C05695	C05697
C05698	C05699	C05703	C05711	C05714	C05730	C05844	C06367
C06368	C06451	C06452	C06454	C06455	C06456	C06457	C06459
C06735	C08325	C14179	C16074	C16075	C16562	C16563	C16564
C16565	C16566	C16567	C17940	C17942	C17944	C17946	C17947
C17949	C17951	C17952	C17962				

(II) 44 enzymes

YBL064C	YBR244W	YBR263W	YDL066W	YDR242W
YDR321W	YDR453C	YER070W	YER073W	YGR007W
YGR019W	YGR180C	YGR202C	YGR256W	YHR123W
YHR183W	YIL010W	YIL066C	YIL145C	YIR037W
YJL026W	YJL101C	YKL026C	YKL184W	YKL215C
YLR058C	YLR109W	YLR146C	YLR155C	YLR157C
YLR158C	YLR160C	YLR174W	YLR299W	YML028W
YMR250W	YNL009W	YNL130C	YNL241C	YOL049W
YOR374W	YPL061W	YPL091W	YPR069C	

7. 65 samples in “Glycan Biosynthesis and Metabolism”

(I) 47 small molecules

C00009	C00033	C00043	C00053	C00054	C00096	C00105	C00110
C00133	C00154	C00381	C00621	C00692	C00993	C01050	C01112
C01187	C01212	C01246	C03862	C04121	C04478	C04574	C04631
C04652	C04738	C04824	C04877	C04882	C04919	C04932	C05382
C05892	C05897	C05898	C06022	C06024	C06025	C06026	C06251
C06397	C06398	C07836	C07838	C11472	C17556	C17558	

(II) 18 enzymes

YBL082C	YBR070C	YBR110W	YBR229C	YBR243C
YGL022W	YGL027C	YGL047W	YGL065C	YGL156W
YGR036C	YJR131W	YMR013C	YMR281W	YNR030W
YPL175W	YPL227C	YPR183W		

8. 437 samples in “Metabolism of Cofactors and Vitamins”

(I) 350 small molecules

C00001	C00003	C00006	C00007	C00010	C00016	C00018	C00022
C00025	C00026	C00032	C00037	C00044	C00047	C00049	C00054
C00061	C00068	C00082	C00097	C00099	C00101	C00106	C00111
C00118	C00120	C00122	C00133	C00141	C00143	C00153	C00156
C00163	C00183	C00188	C00194	C00199	C00217	C00223	C00232
C00234	C00250	C00251	C00253	C00255	C00266	C00268	C00272
C00279	C00314	C00341	C00376	C00378	C00399	C00415	C00429
C00430	C00440	C00445	C00455	C00472	C00473	C00474	C00486
C00500	C00504	C00522	C00530	C00534	C00541	C00544	C00568
C00627	C00647	C00664	C00725	C00748	C00777	C00811	C00828
C00831	C00847	C00853	C00857	C00864	C00882	C00885	C00899
C00900	C00921	C00922	C00931	C00966	C00971	C00992	C01004
C01020	C01024	C01037	C01051	C01056	C01059	C01063	C01079
C01081	C01092	C01134	C01179	C01185	C01215	C01217	C01230
C01268	C01270	C01279	C01297	C01300	C01304	C01352	C01384
C01596	C01727	C01847	C01894	C01909	C01967	C02059	C02094
C02110	C02139	C02191	C02295	C02463	C02469	C02477	C02483
C02588	C02642	C02656	C02691	C02730	C02800	C02823	C02880
C02892	C02918	C02930	C02949	C03028	C03043	C03056	C03114
C03150	C03160	C03194	C03263	C03373	C03393	C03455	C03458
C03479	C03492	C03516	C03541	C03652	C03657	C03672	C03684
C03722	C03741	C03885	C04039	C04079	C04122	C04145	C04146
C04226	C04244	C04294	C04327	C04332	C04352	C04454	C04536
C04556	C04604	C04732	C04752	C04773	C04778	C04807	C04874
C04895	C05200	C05306	C05307	C05313	C05380	C05427	C05552
C05766	C05767	C05768	C05769	C05770	C05772	C05773	C05774
C05775	C05777	C05778	C05786	C05787	C05789	C05790	C05791
C05793	C05794	C05795	C05797	C05798	C05802	C05804	C05805
C05807	C05809	C05810	C05811	C05812	C05813	C05814	C05815

C05817	C05818	C05840	C05841	C05842	C05843	C05847	C05848
C05856	C05912	C05913	C05921	C05924	C05925	C05926	C05944
C06051	C06052	C06054	C06055	C06056	C06178	C06319	C06320
C06399	C06406	C06407	C06408	C06416	C06503	C06504	C06505
C06506	C06507	C06508	C06509	C06510	C06986	C07335	C09332
C10385	C10793	C11061	C11242	C11355	C11437	C11538	C11539
C11540	C11542	C11543	C11545	C11630	C11638	C11829	C11830
C11831	C11832	C11850	C11851	C12147	C13309	C13425	C14151
C14152	C14818	C14819	C15492	C15493	C15523	C15547	C15556
C15563	C15670	C15672	C15809	C15882	C15883	C15986	C15987
C16150	C16151	C16159	C16238	C16242	C16243	C16244	C16390
C16519	C16541	C16677	C16678	C16680	C16681	C16682	C16683
C16999	C17018	C17401	C17432	C17551	C17554	C17561	C17562
C17568	C17569	C17570	C18021	C18022	C18064	C18098	C18131
C18133	C18134	C18154	C18155	C18157	C18239		

(II) 87 enzymes

YAR071W	YBL013W	YBL033C	YBR035C	YBR084W
YBR092C	YBR093C	YBR145W	YBR153W	YBR176W
YBR213W	YBR256C	YBR263W	YCL009C	YCL017C
YCR053W	YDL024C	YDL045C	YDL141W	YDL168W
YDL205C	YDR019C	YDR044W	YDR047W	YDR196C
YDR232W	YDR236C	YDR402C	YDR408C	YDR481C
YDR487C	YDR531W	YEL029C	YER014W	YER183C
YFR047C	YGL037C	YGL040C	YGL125W	YGL245W
YGL256W	YGR010W	YGR204W	YGR267C	YGR286C
YHR063C	YHR074W	YHR208W	YHR215W	YIL145C
YJL046W	YJR016C	YJR148W	YKR069W	YKR080W
YLR028C	YLR058C	YLR209C	YLR239C	YLR328W
YLR355C	YMR083W	YMR108W	YMR120C	YMR303C
YNL256W	YNR027W	YNR033W	YNR057C	YNR058W
YOL033W	YOL055C	YOL086C	YOL096C	YOR074C
YOR143C	YOR176W	YOR184W	YOR196C	YOR209C
YOR236W	YOR278W	YPL023C	YPL214C	YPL258C
YPR073C	YPR127W			

9. 525 samples in “Metabolism of Terpenoids and Polyketides”

(I) 507 small molecules

C00002	C00008	C00015	C00019	C00020	C00022	C00024	C00029
C00033	C00061	C00082	C00083	C00100	C00103	C00118	C00129
C00136	C00147	C00170	C00187	C00190	C00196	C00235	C00251
C00332	C00341	C00353	C00356	C00371	C00400	C00418	C00448
C00477	C00521	C00553	C00621	C00630	C00688	C00744	C00805
C00842	C00843	C00859	C00885	C00964	C00979	C01033	C01107
C01123	C01126	C01143	C01164	C01179	C01230	C01414	C01433

C01457	C01499	C01500	C01512	C01513	C01661	C01699	C01767
C01789	C01804	C01841	C01847	C01848	C01852	C01860	C01907
C01912	C01920	C01946	C02004	C02029	C02034	C02035	C02094
C02344	C02400	C02452	C02462	C02485	C02513	C02557	C02576
C02633	C02811	C03069	C03190	C03198	C03220	C03240	C03319
C03423	C03461	C03579	C03590	C04083	C04145	C04146	C04171
C04216	C04268	C04435	C04574	C04713	C05413	C05414	C05427
C05430	C05431	C05432	C05433	C05434	C05435	C05821	C05859
C06069	C06071	C06074	C06076	C06082	C06087	C06088	C06089
C06090	C06091	C06092	C06093	C06094	C06095	C06098	C06099
C06111	C06307	C06308	C06394	C06570	C06571	C06616	C06624
C06627	C06630	C06633	C06634	C06635	C06654	C06691	C06769
C06801	C07271	C07276	C07277	C07330	C07394	C07667	C08579
C08583	C08584	C08585	C08586	C08590	C08591	C08592	C08601
C08606	C08614	C08814	C09094	C09118	C09183	C09190	C09621
C09629	C09665	C09666	C09672	C09684	C09694	C09699	C09704
C09707	C09723	C09769	C09844	C09847	C09848	C09849	C09871
C09880	C09884	C09893	C11383	C11384	C11386	C11388	C11389
C11393	C11395	C11398	C11402	C11403	C11405	C11406	C11407
C11408	C11409	C11410	C11411	C11412	C11413	C11414	C11415
C11416	C11417	C11418	C11419	C11421	C11434	C11435	C11436
C11437	C11453	C11636	C11672	C11673	C11700	C11811	C11853
C11854	C11855	C11857	C11858	C11859	C11860	C11861	C11862
C11863	C11864	C11865	C11867	C11868	C11869	C11870	C11871
C11872	C11873	C11874	C11875	C11878	C11879	C11880	C11881
C11882	C11883	C11887	C11889	C11893	C11894	C11895	C11897
C11900	C11901	C11902	C11907	C11908	C11911	C11912	C11916
C11918	C11920	C11921	C11924	C11933	C11938	C11939	C11940
C11941	C11942	C11943	C11944	C11945	C11946	C11950	C11951
C11952	C11967	C11971	C11983	C11989	C11990	C11993	C11994
C11995	C11996	C11997	C12000	C12002	C12014	C12026	C12037
C12038	C12044	C12077	C12106	C12107	C12108	C12109	C12142
C12176	C12210	C12212	C12213	C12214	C12215	C12216	C12219
C12220	C12221	C12246	C12247	C12319	C12321	C12323	C12324
C12325	C12366	C12367	C12368	C12369	C12370	C12371	C12376
C12378	C12379	C12381	C12382	C12383	C12384	C12387	C12388
C12389	C12390	C12391	C12392	C12393	C12395	C12396	C12397
C12398	C12399	C12400	C12401	C12402	C12404	C12406	C12412
C12413	C12414	C12415	C12416	C12417	C12419	C12420	C12421
C12422	C12423	C12424	C12425	C12426	C12427	C12428	C12429
C12430	C12432	C12434	C12435	C12436	C12451	C13273	C13431
C13433	C13453	C13454	C13455	C13456	C14540	C14721	C14722
C15514	C15545	C15546	C15784	C15785	C15786	C15787	C15788
C15789	C15790	C15791	C15792	C15793	C15794	C15795	C15797

C15798	C15799	C15800	C15801	C15802	C15803	C15859	C15860
C15870	C15874	C15877	C15878	C15881	C15885	C15886	C15888
C15892	C15895	C15898	C15900	C15902	C15903	C15908	C15943
C15965	C15966	C15967	C15968	C15969	C15970	C15971	C15981
C16028	C16141	C16142	C16143	C16144	C16145	C16146	C16147
C16148	C16251	C16269	C16270	C16276	C16280	C16282	C16284
C16286	C16291	C16340	C16427	C16430	C16431	C16441	C16447
C16448	C16449	C16461	C16462	C16464	C16470	C16493	C16494
C16495	C16496	C16497	C16499	C16500	C16502	C16503	C16504
C16521	C16523	C16826	C16829	C17277	C17432	C17621	C17622
C17733	C17735	C17953	C17954	C18015	C18025	C18037	C18211
C18221	C18222	C18223	C18224	C18225	C18226	C18227	C18228
C18229	C18295	C18297					

(II) 18 enzymes

YBR117C	YER073W	YIL160C	YJL167W	YLR450W
YML075C	YML126C	YMR208W	YMR220W	YNR016C
YNR043W	YOR274W	YOR374W	YPL028W	YPL061W
YPL069C	YPL117C	YPR074C		

10. 526 samples in “Biosynthesis of Other Secondary Metabolites”

(I) 509 small molecules

C00002	C00025	C00031	C00043	C00047	C00048	C00061	C00062
C00073	C00078	C00079	C00082	C00092	C00097	C00103	C00108
C00118	C00123	C00132	C00134	C00137	C00141	C00148	C00166
C00183	C00223	C00233	C00253	C00254	C00315	C00323	C00355
C00385	C00389	C00398	C00406	C00407	C00408	C00411	C00413
C00423	C00450	C00463	C00482	C00483	C00509	C00540	C00590
C00616	C00671	C00688	C00691	C00729	C00745	C00757	C00761
C00783	C00786	C00811	C00814	C00822	C00842	C00852	C00858
C00903	C00916	C00933	C00956	C00974	C01091	C01121	C01138
C01179	C01180	C01197	C01214	C01221	C01263	C01265	C01283
C01294	C01298	C01378	C01416	C01441	C01456	C01460	C01470
C01477	C01479	C01494	C01514	C01516	C01527	C01533	C01562
C01610	C01617	C01672	C01701	C01709	C01735	C01737	C01743
C01745	C01746	C01752	C01759	C01761	C01762	C01772	C01795
C01847	C01851	C01852	C02046	C02066	C02074	C02105	C02106
C02134	C02151	C02153	C02325	C02495	C02621	C02627	C02646
C02666	C02673	C02675	C02723	C02887	C02890	C02906	C02916
C02920	C02937	C02947	C02954	C03005	C03112	C03219	C03239
C03319	C03329	C03442	C03470	C03506	C03515	C03567	C03582
C03648	C03719	C03758	C03765	C03882	C03951	C04006	C04043
C04076	C04092	C04118	C04271	C04280	C04293	C04353	C04443
C04444	C04608	C04673	C04767	C04831	C04858	C05080	C05158
C05174	C05175	C05176	C05177	C05178	C05179	C05189	C05191

C05193 C05194 C05202 C05220 C05243 C05315 C05316 C05376
C05556 C05557 C05587 C05604 C05607 C05608 C05610 C05619
C05623 C05625 C05627 C05631 C05837 C05838 C05851 C05901
C05903 C05904 C05905 C05906 C05907 C05908 C05909 C05911
C06160 C06161 C06162 C06165 C06167 C06171 C06172 C06173
C06174 C06175 C06176 C06177 C06178 C06179 C06180 C06181
C06182 C06183 C06185 C06224 C06327 C06366 C06511 C06516
C06520 C06521 C06524 C06533 C06561 C06562 C06563 C06564
C06565 C06566 C06568 C06569 C06655 C06656 C06657 C06658
C06659 C06660 C06661 C06662 C07026 C07031 C07032 C07130
C07201 C07204 C07304 C07307 C07475 C07480 C07481 C07592
C07593 C07756 C08401 C08409 C08412 C08417 C08426 C08526
C08532 C08537 C08538 C08539 C08540 C08543 C08544 C08552
C08557 C08565 C08578 C08604 C08620 C08639 C08641 C08650
C08725 C09024 C09099 C09126 C09239 C09244 C09248 C09320
C09328 C09330 C09337 C09390 C09421 C09581 C09614 C09727
C09751 C09756 C09762 C09789 C09806 C09826 C09827 C10028
C10044 C10098 C10107 C10126 C10171 C10173 C10192 C10200
C10205 C10208 C10216 C10434 C10462 C10502 C10503 C10509
C10516 C10520 C10521 C10522 C10538 C10632 C10738 C10773
C10783 C10850 C10858 C10860 C10865 C10945 C11130 C11250
C11351 C11620 C11632 C11633 C11634 C11635 C11641 C11643
C11675 C11676 C11677 C11678 C11679 C11680 C11682 C11783
C11807 C11808 C11809 C11810 C11812 C11813 C11814 C11816
C11817 C11907 C12032 C12092 C12093 C12096 C12123 C12125
C12127 C12128 C12134 C12136 C12137 C12138 C12139 C12140
C12141 C12162 C12173 C12203 C12204 C12205 C12206 C12208
C12249 C12312 C12440 C12449 C12452 C12453 C12454 C12455
C12456 C12457 C12458 C12468 C12473 C12474 C12476 C12477
C12480 C12626 C12631 C12633 C12634 C12640 C12643 C12644
C12646 C12647 C13747 C14314 C14536 C15509 C15525 C15530
C15567 C15612 C15666 C15769 C15770 C15772 C16075 C16187
C16190 C16191 C16192 C16195 C16197 C16222 C16223 C16225
C16226 C16228 C16229 C16230 C16232 C16299 C16303 C16306
C16315 C16352 C16353 C16355 C16356 C16357 C16358 C16359
C16360 C16361 C16362 C16363 C16364 C16365 C16366 C16404
C16406 C16416 C16417 C16418 C16419 C16422 C16700 C16709
C16710 C16712 C17209 C17213 C17217 C17221 C17235 C17239
C17245 C17246 C17250 C17254 C17580 C17581 C17582 C17585
C17591 C17592 C17742 C17743 C17746 C17749 C17750 C17753
C17754 C17755 C17756 C17757 C18004

(II) 17 enzymes

YCL040W YDR287W YDR402C YFR053C YGL202W
YGL253W YHR046C YHR137W YHR208W YIL116W

YJL153C YJR148W YKL106W YKL127W YLR027C
YMR105C YMR278W

11. 730 samples in “Xenobiotics Biodegradation and Metabolism”
(I) 709 small molecules

C00011	C00022	C00024	C00036	C00042	C00048	C00058	C00067
C00084	C00086	C00090	C00091	C00108	C00122	C00146	C00156
C00160	C00163	C00164	C00180	C00186	C00196	C00209	C00222
C00230	C00237	C00261	C00292	C00332	C00414	C00472	C00479
C00511	C00512	C00527	C00530	C00544	C00556	C00568	C00571
C00587	C00596	C00601	C00628	C00632	C00633	C00682	C00755
C00805	C00827	C00829	C00846	C00854	C00870	C00877	C00894
C01010	C01036	C01061	C01063	C01108	C01144	C01147	C01163
C01198	C01273	C01278	C01380	C01407	C01424	C01454	C01455
C01467	C01468	C01471	C01504	C01516	C01542	C01548	C01566
C01606	C01659	C01751	C01880	C01983	C01984	C01987	C01998
C02083	C02124	C02137	C02183	C02222	C02235	C02236	C02247
C02271	C02274	C02357	C02359	C02364	C02370	C02371	C02372
C02375	C02378	C02380	C02411	C02480	C02501	C02505	C02519
C02526	C02575	C02617	C02625	C02720	C02734	C02802	C02814
C02824	C02909	C02923	C02933	C02949	C03012	C03057	C03067
C03077	C03164	C03198	C03203	C03223	C03233	C03351	C03360
C03434	C03453	C03569	C03572	C03585	C03586	C03589	C03590
C03664	C03671	C03676	C03739	C03824	C03918	C03925	C04091
C04112	C04115	C04178	C04242	C04314	C04324	C04431	C04434
C04451	C04468	C04484	C04522	C04553	C04558	C04559	C04592
C04596	C04623	C04646	C04706	C04729	C04783	C05011	C05165
C05361	C05375	C05593	C05616	C05618	C05852	C06002	C06033
C06102	C06103	C06104	C06105	C06174	C06202	C06203	C06204
C06205	C06206	C06210	C06317	C06318	C06321	C06322	C06328
C06329	C06333	C06335	C06336	C06337	C06387	C06547	C06548
C06551	C06552	C06553	C06554	C06555	C06556	C06557	C06558
C06559	C06560	C06575	C06576	C06577	C06578	C06579	C06580
C06581	C06582	C06584	C06588	C06589	C06593	C06594	C06596
C06597	C06598	C06599	C06600	C06601	C06602	C06603	C06604
C06605	C06606	C06607	C06608	C06609	C06610	C06611	C06612
C06613	C06614	C06636	C06637	C06638	C06639	C06640	C06641
C06642	C06643	C06647	C06648	C06650	C06670	C06671	C06672
C06673	C06674	C06675	C06676	C06677	C06678	C06679	C06680
C06711	C06712	C06714	C06715	C06719	C06723	C06728	C06730
C06749	C06752	C06753	C06754	C06755	C06756	C06757	C06758
C06760	C06762	C06789	C06790	C06791	C06792	C06793	C06813
C06837	C06868	C06876	C06899	C06988	C06989	C06990	C07047
C07054	C07073	C07075	C07083	C07084	C07085	C07086	C07088

C07089 C07090 C07091 C07092 C07093 C07094 C07095 C07096
C07097 C07098 C07099 C07100 C07101 C07102 C07103 C07108
C07111 C07113 C07114 C07118 C07163 C07185 C07189 C07208
C07209 C07211 C07212 C07213 C07214 C07215 C07216 C07446
C07447 C07490 C07492 C07493 C07495 C07496 C07501 C07535
C07557 C07572 C07585 C07643 C07644 C07645 C07646 C07647
C07648 C07649 C07715 C07720 C07723 C07727 C07728 C07729
C07731 C07732 C07888 C08012 C08060 C08061 C08063 C08733
C08734 C08735 C08737 C09811 C09812 C09813 C09814 C09815
C09816 C09817 C09818 C09819 C09820 C09821 C09822 C09823
C10700 C10833 C11004 C11006 C11036 C11057 C11088 C11148
C11149 C11150 C11173 C11249 C11272 C11348 C11376 C11422
C11427 C11429 C11430 C11432 C11433 C11519 C11520 C11713
C11714 C11736 C11785 C12313 C12650 C12673 C12739 C12831
C12833 C12834 C12835 C12836 C12837 C12838 C13620 C13621
C13622 C13623 C13624 C13625 C13626 C13628 C13629 C13631
C13632 C13633 C13634 C13635 C13636 C13637 C13638 C14039
C14040 C14082 C14088 C14089 C14090 C14091 C14093 C14096
C14097 C14098 C14099 C14101 C14103 C14110 C14111 C14113
C14114 C14115 C14116 C14117 C14120 C14143 C14144 C14145
C14147 C14220 C14298 C14315 C14335 C14401 C14418 C14419
C14450 C14453 C14519 C14556 C14573 C14602 C14604 C14610
C14783 C14785 C14786 C14787 C14788 C14789 C14790 C14839
C14840 C14841 C14842 C14843 C14844 C14849 C14850 C14851
C14852 C14853 C14854 C14857 C14858 C14859 C14862 C14863
C14864 C14866 C14867 C14868 C14869 C14870 C14871 C14872
C14873 C14875 C14876 C14877 C16074 C16075 C16181 C16182
C16193 C16194 C16199 C16200 C16201 C16202 C16204 C16206
C16207 C16208 C16209 C16211 C16212 C16213 C16215 C16234
C16235 C16246 C16247 C16248 C16262 C16266 C16267 C16268
C16272 C16273 C16281 C16283 C16348 C16391 C16392 C16393
C16394 C16395 C16396 C16397 C16398 C16399 C16400 C16401
C16402 C16411 C16412 C16413 C16472 C16473 C16474 C16475
C16476 C16477 C16544 C16545 C16547 C16548 C16549 C16550
C16551 C16552 C16553 C16554 C16555 C16556 C16557 C16558
C16559 C16560 C16561 C16569 C16570 C16571 C16572 C16577
C16578 C16586 C16587 C16591 C16592 C16595 C16596 C16601
C16602 C16603 C16604 C16607 C16608 C16609 C16610 C16613
C16614 C16617 C16618 C16619 C16621 C16622 C16623 C16630
C16631 C16632 C16633 C16634 C16635 C16641 C16643 C16648
C16649 C16650 C16651 C16652 C16653 C16654 C16655 C16656
C16657 C16658 C16659 C16660 C16661 C16662 C18099 C18100
C18101 C18102 C18103 C18104 C18105 C18106 C18107 C18108
C18109 C18110 C18111 C18112 C18113 C18114 C18115 C18116

C18117 C18118 C18119 C18120 C18121 C18122 C18123 C18180
C18215 C18216 C18236 C18240 C18246 C18247 C18248 C18249
C18250 C18251 C18252 C18253 C18254 C18255 C18257 C18258
C18259 C18260 C18263 C18264 C18269 C18281 C18286 C18300
C18302 C18304 C18305 C18306 C18307 C18308 C18311 C18312
C18314 C18315 C18316 C18317 C18320

(II) 21 enzymes

YBR145W YBR208C YDL086W YDL168W YDR242W
YDR402C YGL256W YHR216W YIL160C YLR245C
YLR432W YML056C YML106W YMR083W YMR169C
YMR170C YMR217W YMR271C YMR303C YNR012W
YOL086C
